# Pannexin1 Channel-Mediated Inflammation in Acute Ischemic Stroke

**DOI:** 10.14336/AD.2023.0303

**Published:** 2024-05-07

**Authors:** Yubing Huang, Yutong Shi, Mengmeng Wang, Bingyi Liu, Xueqin Chang, Xia Xiao, Huihui Yu, Xiaodie Cui, Ying Bai

**Affiliations:** ^1^Department of Neurology, Dalian University Affiliated Xinhua Hospital, Dalian, Liaoning, China; ^2^Medical College, Institute of Microanalysis, Dalian University, Dalian, Liaoning, China; ^3^Graduate School, Dalian University, Dalian, Liaoning, China

**Keywords:** pannexin1, Acute ischemic stroke, inflammation, NLRP3 inflammasome, Brain-organoid-on-a-chip, exosomal miRNAs

## Abstract

Emerging evidence suggests that inflammation mediated by the pannexin1 channel contributes significantly to acute ischemic stroke. It is believed that the pannexin1 channel is key in initiating central system inflammation during the early stages of acute ischemic stroke. Moreover, the pannexin1 channel is involved in the inflammatory cascade to maintain the inflammation levels. Specifically, the interaction of pannexin1 channels with ATP-sensitive P2X7 purinoceptors or promotion of potassium efflux mediates the activation of the NLRP3 inflammasome, triggering the release of pro-inflammatory factors such as IL-1 and IL-18, exacerbating and sustaining inflammation of brain. Also, increased release of ATP induced by cerebrovascular injury activates pannexin1 in vascular endothelial cells. This signal directs peripheral leukocytes to migrate into ischemic brain tissue, leading to an expansion of the inflammatory zone. Intervention strategies targeting pannexin1 channels may greatly alleviate inflammation after acute ischemic stroke to improve this patient population's clinical outcomes. In this review, we sought to summarize relevant studies on inflammation mediated by the pannexin1 channel in acute ischemic stroke and discussed the possibility of using brain organoid-on-a-chip technology to screen miRNAs that exclusively target the pannexin1 channel to provide new therapeutic measures for targeted regulation of pannexin1 channel to reduce inflammation in acute ischemic stroke.

## 1. Introduction

Stroke is the second leading cause of death and disability in people over 60 worldwide, significantly burdening the healthcare systems and the global economy [1]. Strokes are generally classified according to etiology as hemorrhagic or ischemic. In both types, blood flow to various brain areas is disrupted, resulting in insufficient blood supply and cerebrovascular destruction events [2, 3]. Risk factors of stroke include sex, infection, vascular malformations, underlying diseases such as hypertension, diabetes, and atrial fibrillation, and external factors related to daily life, like obesity, smoking and alcohol consumption [4]. Current evidence suggests that age has become a major risk factor for stroke, given that substantial advances in medical technology have prolonged human life expectancy [5]. In 2050, the global population over 65 is estimated to exceed 1.5 billion, and stroke is expected to increasingly affect the world in the future [6]. In this respect, ischemic stroke reportedly accounts for more than 80% of all strokes, and only two effective treatments are available for acute ischemic stroke: intravenous thrombolysis with tissue plasminogen activator (tPA) and intravascular mechanical thrombectomy. Both approaches have narrow time windows that offer little patient benefit [7-9]. These findings suggest that new and effective methods are urgently needed to improve the prognosis of this patient population.

Ischemic stroke caused by blood clots or other emboli blocking the blood vessel causes dramatic changes in the physiological state of the brain tissue. During this process, the massive reduction of adenosine triphosphate (ATP) leads to the release of excitatory amino acids, causing excitotoxic damage to neurons [10]. In addition, ATP depletion causes paralysis of the Na^+^/K^+^ pump, leading to increased extracellular K^+^ levels and glutamate release [11, 12]. Glutamate is a key factor affecting the diffusion around infarction [13, 14]. On the one hand, high extracellular glutamate levels stimulate Na^+^/ Ca2^+^ channels to pair with N-methyl-D-aspartate receptors (NMDAR), increasing Na^+^ and Cl^-^ levels and causing passive water inflow, leading to cytotoxic edema [15, 16]. Meanwhile, continuous activation of NMDAR has been reported to significantly increase intracellular Ca^2+^ levels [17]. On the other hand, extracellular glutamate also reportedly activates N-methyl-D-aspartic acid and α-amino-3-hydroxy-5-methyl-4-isooxazopropionic acid, which significantly increases intracellular Ca^2+^ levels [13]. The synergistic effect of receptors and voltage-gated Ca2+ channels leads to mitochondrial calcium overload, which further affects ATP production. These parallel events, including energy expenditure and ion imbalance, trigger a cascade of inflammatory responses that play an important role in the development and prognosis of ischemic stroke. Interestingly, pannexins, transmembrane channels that connect the inner and outer spaces of cells, play an important role in cellular stress responses to ischemia and hypoxia [18]. In particular, the pannexin1(PANX1) channel is a unique member of the pannexins family widely associated with increased inflammation during the acute phase of ischemic stroke [19]. The pro-inflammatory effect of the pannexin1 channel in acute ischemic stroke is mainly related to its ATP-sensitive mechanical pathway [20]. ATP is an important substance for signal communication between microglia and astrocytes, and ATP externalization is a key step in activating the NLRP3 inflammasome to produce inflammatory cytokine IL-1β after ischemic stroke [21-23]. Inhibition of the PANX1 channel may yield anti-inflammatory effects, making it a potential therapeutic target for acute ischemic stroke.

### 1.1 Inflammation and acute ischemic stroke

Inflammation is the body's complex biological response to danger signals such as chemical (acids and bases), physical (ionizing radiation, ultrasound) and biological factors (bacteria, viruses, fungi, exotoxins and endotoxins), and is a leading mediator of cell and tissue damage resulting in many human diseases [24]. Within nervous tissues, inflammation refers to vascular events that trigger innate immune responses in the central and peripheral nervous system during pathological events after injury by infectious or non-infectious factors [25, 26]. Inflammation has become a potential target for the treatment of ischemic stroke, given its involvement in the pathogenesis of ischemic stroke. Current evidence suggests the presence of the inflammatory response throughout the ischemic stroke process, including the acute, developmental, and rehabilitation stages [27, 28]. Microglia and astrocytes are the first to respond to the danger signal during an ischemic stroke. They are activated rapidly within minutes to hours to secrete many pro-inflammatory factors such as IL-1β, Interleukin 6 (IL-6), Interleukin 18 (IL-18), tumor necrosis factor α(TNF-α), matrix metalloproteinases (MMPs), ATP and inducible Nitric Oxide Synthase iNOS [29]. In this regard, ATP released by injured cells acts as a damage associated molecular pattern (DAMP) to signal danger, which further activates glial cells and recruits peripheral effector cells to converge on the lesion [30]. Subsequently, peripheral inflammatory cells such as neutrophils, macrophages, and lymphocytes are recruited into the ischemic area within hours to days due to changes in cell adhesion molecules on endothelial cells [31, 32]. The infiltration of inflammatory cells produces more pro-inflammatory factors and MMPs, such as MMP-9 [33]. In addition, excessive production of Reactive oxygen species(ROS) caused by the accumulation of neutrophils exacerbates nerve injury after stroke and promotes further development of the inflammatory cascade reaction [34]. Although it has been suggested that inflammatory responses are beneficial for neurological repair in the later stages of ischemic stroke, it is generally believed that early inflammatory responses can exacerbate brain damage. Consistently, early implementation of anti-inflammatory measures, including anti-leukocyte or anti-adhesion molecule strategies and anti-inflammatory factor strategies, has been shown to reduce ischemic injury in animal models of ischemic stroke [29, 35].

### 1.2 The pannexins family

The pannexins family of genes (from Greek, Latin pan -"all, throughout" and nexus- "connection, bond", abbreviated PANX) was discovered by Panchin and his colleagues at the beginning of the 21st century [36]. Pannexin1 (PANX1) was the first identified member. Pannexin2 (PANX2) and a third family member, pannexin3 (PANX3), have been subsequently recognized. Based on the similarity between the lower invertebrate chordates pannexins and innexins, it has been speculated that there is an evolutionary relationship between them. Therefore, pannexins have long been considered interstitial junction-forming proteins in invertebrates [36-38]. However, there is an increasing consensus that pannexin is neither a gap junction nor an intercellular channel on adjacent membranes [39-41]. Sosinsky et al. thereby proposed the concept of pannexins as channels in 2011 [42].

Pannexins are universally expressed in the human body, including the eyes, thyroid, prostate, kidney, liver, and central nervous system [43]. Current evidence suggests that PANX1 is widely expressed in almost all tissues of mammals and is the most characteristic of the three pannexins currently present in rodent and human genomes [44]. It has been reported that PANX1 is present in lymphocytes, adipocytes, endothelium, epithelial cells, and muscle cells [45-51]. In the CNS, PANX1 has been documented in neurons and glial cells (microglia and astroglia) in the cerebellum, cerebral cortex, thalamus, hippocampus, spinal cord, and Schwann cells [52, 53]. Unlike PANX1, which is widely expressed in the human body, PANX2 is highly expressed in the brain, spinal cord and hippocampus, with limited expression in other tissues, while PANX3 can only be detected in skin and bone tissues [54, 55]. Interestingly, Isakson et al. in 2012 first reported that pannexins were also present in the arterial tree, and PANX1 is the major subtype [56]. Pannexins play an important role in the physiological process of intercellular communication since their opening allows for the selective passage of small ions such as Na+, K+, Ca2+ and ATP [57, 58]. It is worth noting that the PANX1 channel is an important pathway of ATP transmission involved in the occurrence of many central system diseases. In this respect, the PANX1 channel has been reported to contribute to chronic neuropathic pain and ischemic stroke [50, 59].

## 2. The roles of Pannexins in acute ischemic stroke

PANX1 has received increasing attention due to its close association with various diseases associated with the central nervous system, especially for its role in inflammation after ischemic stroke [60, 61]. Although PANX2 has been identified to be associated with some neurological disorders, the function of PANX2 in the central nervous system is not fully understood, because PANX2 is located in the cellular chamber, which limits the evaluation of its channel function by electrophysiological methods [55, 62, 63].

### 2.1 Pannexin1 channel in acute ischemic stroke

The first evidence that PANX1 channels may contribute to ischemic brain injury came from experimental data published by Thompson and colleagues in 2006. They detected activation and opening of large conductance channels in Oxygen-Glucose deprivation (OGD)-induced hippocampal isolated neurons, and currents in these large conductance channels produced hypoxic depolarization, which led to neuronal death. When the non-specific gap junction blocker Carbenoxolone (CBX) was used, the currents showed a sharp decrease, which they attributed to the blocking of PANX1 by CBX [64, 65]. Further study revealed that the PANX1 channel mediates the inflammatory response after stroke [60, 66].

**Table 1:** Key points of inflammation mediated by the PANX1 channel in acute ischemic stroke.

Time	Mechanism
**several minutes**	The activation of PANX1 channels on microglia and astrocytes enables K+ and ATP exosmosis to activate the NLRP3 inflammasome, thus producing pro-inflammatory factors such as IL-1β, IL-18 and TNF-α, preliminatively initiating neuroinflammation
**30 min**	Neutrophils are recruited to gather in the ischemic area in response to the "find me" signal released by ATP, which will produce more pro-inflammatory factors to aggravate the injury of neuroinflammation
**24 h**	The continuous work of the PANX1-NLRP3 signaling axis allows IL-1β and IL-18 proinflammatory factors to peak and maintain the neuroinflammatory response

We summarize the key events of inflammation mediated by PANX1 channels in acute ischemic stroke (Table 1). As mentioned above, activation of microglia and astrocytes represent the first line of defense during the inflammatory response following an acute ischemic stroke [29, 67, 68]. It has been observed that microglia cells could be rapidly activated and differentiated into an M1 or M2 phenotype to adapt to different kinds of stimuli. However, these two different types of microglia served completely different roles in physiology and function following ischemic stroke [69]. For example, activation of M1 microglia was identified to act as a pro-inflammatory role, which resulted in brain damage, while M2 microglia were regarded as an anti-inflammatory state and benefited brain repair [70, 71]. Like microglia, astrocytes have been subject to debate for their potential adverse or advantageous effects during the acute phase of ischemic stroke [72]. However, astrocytes reacted to hyperplasia and released pro-inflammatory molecules and ROS, which were responsible for neuroinflammation and nerve cell death. On the bright side, astrocyte proliferation can form glial scars that limit neuroinflammation and isolate the injury site to enhance neuroprotection [73-75]. As stated, PANX1 channels are thought to be closely related to the cellular interactions between microglia, astrocytes, and neurons [42-44]. During the acute phase of ischemic stroke, PANX1 channels in microglia and astrocytes are activated to release ATP extracellularly to produce various pro-inflammatory factors such as IL-1 and IL-18 [19]. In addition, ATP release from the PANX1 channels may prompt damaged cells to release a "find me" signal early in death to recruit phagocytes [20]. Accordingly, during acute ischemic stroke, ATP released from ischemic neurons recruits peripheral immune cells such as neutrophils, macrophages, and lymphocytes, producing more pro-inflammatory cytokines to converge on ischemic lesions and exacerbate inflammatory damage in ischemic brain tissue [76, 77]. It is worth mentioning that cell death mediated by the PANX1 channel may be pyroptosis. In a study reported by Chen et al. in 2019, where a novel Gsdmd D88A knock-in mouse model was established, it was demonstrated that PANX1 but not GSDMD or GSDME promotes activation of NLRP3 inflammasome and thus induces pyroptosis [78].

Recent studies suggest that using PANX1 channel blockers promotes the survival of stroke neurons and oligodendrocytes and the protection of cerebral white matter, mainly attributed to the inflammatory response mitigation by inhibiting the PANX1 channel [79-81]. In 2012, Bargiotas et al. reported a study using wild-type mice and pannexin double knockout mice (Px1-/- Px2-/-) after implementing a distal middle cerebral artery occlusion (MCAO) for 48 hours. They found less impairment in parameters such as exploration, sensorimotor function, anxiety and behavioral symmetry in pannexin double knockout mice [82]. Freitas-andrade et al. compared the infarct size and the activation of astrocytes and microglia in wild-type and PANX1 knockout mice after middle cerebral artery occlusion in 2017. The infarct size was reduced by approximately 50% in female mice but not in male PANX1 knockout mice due to decreased peri-infarct inflammation and astrocyte responsiveness. The difference in ischemic stroke outcomes associated with gender may have been caused by higher levels of PANX1 channels in female mice than in male mice, while the deletion of PANX1 channels in females considerably countered the deleterious effects of PANX1 channels [83]. Similar results were observed in an experiment that selectively knocked out the PANX1 gene in mouse blood vessels to investigate the effect of PANX1 on cerebral ischemia/reperfusion injury. In this experiment, they substantiated that endothelial cell deletion of the PANX1 gene could significantly reduce inflammation and myogenic tone formation to alleviate cerebral ischemic injury [84].

### 2.2 Activation mechanisms of pannexin1 channel in acute ischemic stroke

Several doctrines may explain the activation mechanism of the PANX1 channel. The first and most classical one proposed by Weilinger et al. suggests that PANX1 channels are recruited in response to NMDAR signaling [85]. NMDAR is a compound composed of four subunits, two of which are basic GluN1 subunits, and the remaining two are derived from GluN2a, GluN2b, GluN2c or GluN2d[85]. Numerous pivotal experiments have demonstrated a strong association between NMDAR activation and PANX1 channels in neurons. Ample literature substantiates that the activation of NMDAR initiates an increase in intracellular Src family kinases (SFKs) that may interact with Y308 at the PANX1 C-terminus [86, 87]. Application of the SFKs antagonist PP2 prevented activation of PANX1 channels in hippocampal slices exposed to hypoxia. Besides, it has been reported that this relationship may be observed throughout the central nervous system [88]. It is highly conceivable that NMDAR modulates PANX1 channels after acute ischemic stroke because of the increased activity of SFKs in hippocampal neurons during ischemic injury [89]. Moreover, mechanical stimuli such as mechanical force and pH are involved in the action of PANX1 channels, which have also been described in different cell types, such as erythrocytes and capillary endothelial cells [78]. Besides, experimental evidence suggests that PANX1 channels can be regulated by ions, mainly K^+^ and Ca^2+^. In a study of PANX1 channels in Xenopus laevis oocytes, the opening of PANX1 channels was regulated by the extracellular K^+^ concentration. In short, increasing extracellular K+ levels could activate PANX1 channels [90]. Similar results were obtained by another group, which concluded that high extracellular K^+^ attenuates the inhibition of PANX1 channel currents [91]. In the central nervous system, healthy neurons and glial cells rely on the Na^+^/K^+^ proton pump to maintain a state of high intracellular K+ and low extracellular K^+^ [92]. There is an increasing consensus that during an acute ischemic stroke, Na^+^/K^+^ pump malfunction leads to rapid reduction and efflux of cell membrane K^+^[93, 94]. The process of K^+^ efflux is accompanied by Ca^2+^ inward flow, causing intracellular Ca^2+^ overload in neuronal and non-neuronal cells [95]. The mechanism of Ca^2+^ activation of PANX1 channels has not been fully elucidated yet. Two mechanisms of Ca^2+^ diffusion have been documented. One is that connexins form gap junctions allowing the flow of the second messenger IP3, facilitating the propagation of calcium waves between neighboring cells [96]. A second possible mechanism is that ATP and purinergic receptors synergistically mediate calcium wave amplification [97, 98]. We hypothesize that Ca^2+^ may synergistically activate PANX1 channels with ATP in acute ischemic stroke, a typical danger signaling molecule regulated at extremely low levels in healthy neuronal cells [99]. When an acute ischemic stroke occurs, the area surrounding the lesion injury releases ATP, which activates purinergic receptors, especially the P2X7 receptor (P2X7R) [56, 100, 101]. Purinergic receptors are highly permeable to Ca^2+^, and the inward flow of Ca^2+^ leads to the opening of PANX1 channels [102, 103].

**Figure 1.**
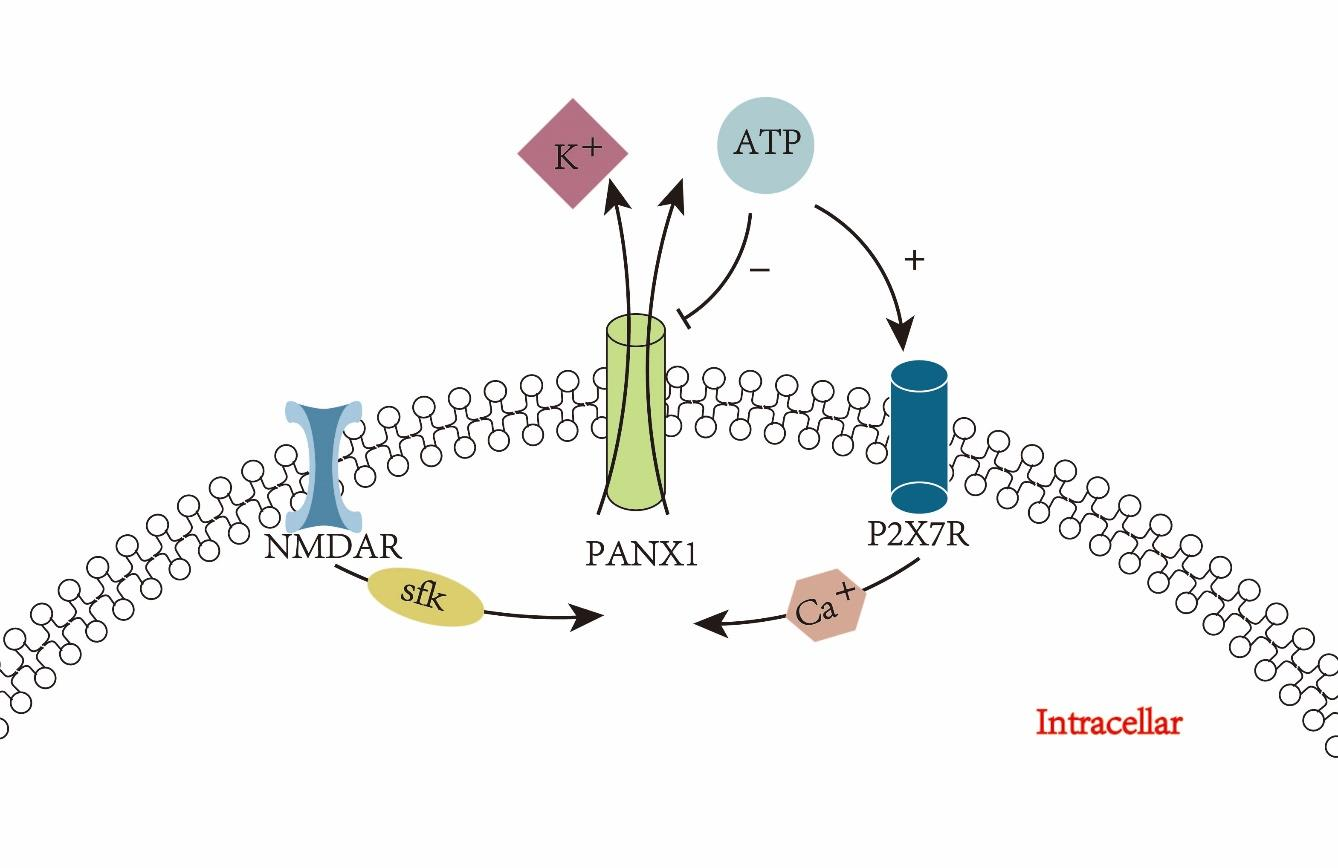
**Elements activating pannexin1**. Increased SFK, potassium outflow, calcium influx, extracellular ATP binding with P2X7 receptor opening pannexin1. High extracellular ATP negative feedback inhibited pannexin1 channel activity.

The putative mechanism of the activation of the PANX1 channel is shown in Figure 1. ATP leaked from the PANX1 channel promotes the permeation of P2X7R to calcium ions, which strongly activates the PANX1 channel. It is highly likely that ATP is continuously released through this positive feedback loop, leading to intense inflammation during cellular ischemia [104]. However, due to the complex stabilization system of the human body, a self-protection mechanism may exist that could prevent (if not stop or counteract) this destructive positive feedback loop. Interestingly, significant experimental evidence substantiates the inhibitory effect of high extracellular ATP concentrations on PANX1 channels [105, 106]. However, during acute ischemic stroke, the convergence of the aforementioned factors, including K+, ATP and hypoxia, overstimulates PANX1 channels and may disable the negative feedback system. The imbalance in the activation system leads to a massive accumulation of ATP, allowing the convergence of pro-inflammatory cells from the center or even the periphery to the injury site, subsequently leading to the death of other healthy cells [107, 108].

### 2.3 The PANX1-NLRP3 signaling axis

**Figure 2.**
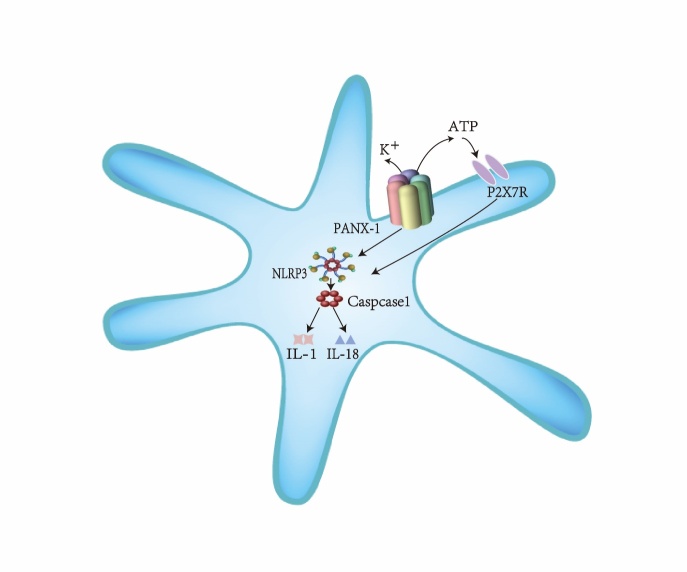
**Pannexin1-NLRP3 signaling axis**. Activation of Pannexin1 activates the downstream NLRP3 inflammasome. Caspace1, which depends on NLRP3 inflammasome activity, is activated to release many inflammatory factors such as IL1β and IL18. Neuroinflammation is amplified and maintained by this signaling cascade.

The PANX1 channel participates in the inflammatory cascade of ischemic stroke relevant to the PANX1-NLRP3 signaling axis (Fig. 2). The NLRP3 inflammasome is a cytoplasmic macromolecular complex composed of the NLRP3 receptor of the NLRP family and procasp-1 [109]. Different stimuli such as RNA, potassium efflux and ATP may be potent activators of the NLRP3 inflammasome [110]. During the first 24 hours after ischemia-reperfusion injury, activation of signaling pathways such as NF-κB and MAPK leads to the upregulation of NLRP3 inflammasomes, which in turn promote the maturation and secretion of large amounts of pro-inflammatory factors, including IL-1 and IL-18 [111-114]. Excess pro-inflammatory factors are widely thought to lead to neuronal death and exacerbate ischemic stroke. In addition, activation of the NLRP3 inflammasome and sustained induction of IL-1 is thought to trigger endothelial cell injury, substantiating the association between thrombosis and inflammatory pathways in acute ischemic stroke [94].

There are two theories regarding NLRP3 inflammasome activation by PANX1 channels. One is that the PANX1 channel activates the NLRP3 inflammasome by activating P2X7R through ATP efflux. In this respect, Niamh Murphy et al. reported that ATP action on P2X7R could effectively activate the NLRP3 inflammasome in glial cells, while knockdown of PANX1 gene inhibits ATP-activated P2X7R-induced IL-1β efflux. This finding suggests that NLRP3 inflammasome activation may be related to ATP/2X7R activation by PANX1 [30]. Cisneros-Mejorado et al. consistently used a P2X7R inhibitor or PANX1 channel blocker to protect mouse neurons from ischemia/hypoxia, yielding similar improvements. This finding substantiated that P2X7R and PANX1 channels are in the same signaling cascade in ischemia-triggered cell death [115]. A second theory suggests that PANX1 channels may drive NLRP3 inflammasome assembly by promoting K+ efflux rather than driving ATP binding to P2X7R. Consistently, Chen et al. found that caspase-1 activation, dependent on NLRP3 inflammasome activity, was unaffected during P2X7-/ - macrophage apoptosis, suggesting that the deletion of the P2X7 gene does not affect NLRP3 inflammasome activity [78]. However, the PANX1 channel is certainly a basic requirement for NLRP3 activation. From this perspective, PANX1 channels form part of the inflammasome.

## 3. Exosomes carrying miRNAs can target the PANX1 channel

So far, dozens of drugs have been reported to inhibit the PANX1 channel, such as CBX, mefloquine and probenecid [116-118]. Although small molecule inhibitors of PANX1 (10panx) can target the PANX1 channel, therapeutic strategies that regulate PANX1 expression at the genetic level remain attractive [119, 120]. With this in mind, we focused on miRNAs, molecules that do not encode in the body but negatively regulate the translation of downstream genes and thus inhibit the synthesis of target proteins. Finding or developing a miRNA that disrupts or strongly inhibits the mRNA translation of PANX1 may be a new research direction. Using the keywords "pannexin1 "and" miRNAs", studies were retrieved from Pubmed and Web of Science databases. Ping Han et al. corroborated that overexpression of miRNA-10a-5p could effectively reduce the expression of PANX1 protein in rat hippocampus, which is expected to become a new target for the treatment of intractable epilepsy [121]. Furthermore, Yokoyama S et al. used doxorubicin-induced amoeboid CD44 high oral cancer cells to examine the role of miR- 224- 5p and found that miR- 224- 5p could affect apoptotic pathways by inhibiting PANX1 channels [122]. Over the years, miRNAs targeting the regulation of PANX1 channels have been largely understudied, and more miRNA molecules remain to be discovered. Accordingly, it is necessary to study the role of these molecules in acute ischemic stroke. In addition, given that a miRNA molecule has multiple downstream target genes, artificial synthesis may be an option if existing miRNAs cannot meet the requirements of highly selective targeting of PANX1 channels.

Considering the role of the blood-brain barrier (BBB) in blocking the penetration of peripheral drugs and the invasive route of direct injection of miRNA into the brain, the use of a carrier that can easily cross the blood-brain barrier to deliver miRNAs from the periphery to the ischemic center represents an ideal treatment scheme. Exosomes represent tiny membrane vesicles about 40 to 100nm in diameter secreted by almost all cells. Exosomes are rich in lipid molecules, which are beneficial for direct fusion with the target cell membrane to release the proteins, mRNA and microRNA, etc. Exosome-loaded drug delivery has the advantages of high delivery efficiency, low immunogenicity and good bio-compatibility, embodying a promising drug delivery route. Importantly, unmodified natural exosomes can escape phagocytosis and cross the BBB, while engineered exosomes, such as brain-targeted peptides, can greatly improve the targeting of exosomes to the brain [123, 124]. Since Alvarez-Erviti et al. first transfected lysosome-associated membrane glycoprotein 2b (Lamp2b) and rabies virus glycoprotein (RVG) to successfully obtain exosomes targeting neuronal cells and brain endothelial cells with high selectivity, the construction of Lamp2b-RVG fusion proteins to produce surface modified exosomes has become the predominant way to obtain brain targeted exosomes [125]. In 2017, RVG-modified exosomes containing miR124 were given peripherally in C57BL/6 mice, and miR124 was successfully transported to the site of cerebral infarction and protected against cerebral ischemia [126]. Importantly, exosomes carrying a miRNA that highly targets the PANX1 channel to treat acute ischemic stroke could reduce inflammation and improve patient outcomes.

## 4. Brain-organoid-on-a-chip screening for miRNAs targeting the PANX1 channel

Two main methods are used in the experimental research of the nervous system: cell culture and animal model construction. These experimental methods, however, cannot fully replicate the pathophysiological processes in humans, and the model for acute ischemic stroke studies is the same. Precisely reproducing brain structure and function to simulate the microenvironment of human acute ischemic stroke has huge application potential, for it would make up for the shortcomings of existing research methods. The development of organoid-on-a-chip technology may make this idea possible. Organoid-on-a-chip is a new technology that combines the organ culture principle of cell induction differentiation with an organ-on-a-chip platform to achieve in vitro organ simulation. It not only has the same advantages as organoids with highly similar genetic and epigenetic characteristics to target organs but also integrates the advantages of controllability and repeatability of organ-on-a-chip technology to simulate and reproduce the human tissue microenvironment as comprehensively as possible [127-130]. Besides, this technology brings many benefits when applied to drug development since it saves time and costs, providing an excellent platform for large-scale drug screening [131].

Although it remains challenging to use the brain-organoid-on-a-chip technology to construct an in vitro model of acute ischemic stroke for PANX1 channel research due to the complexity of the nervous system and technical limitations, the recent breakthroughs in the research of brain-organoid-on-a-chip bring hope. Specifically, the tubular human brain organoid chips proposed by Ao et al. in 2021 can be an alternative model for the preliminary screening of miRNAs that highly target PANX1 channels to lay a foundation for the clinical transformation of PANX1 channels [132]. The miRNAs to be screened have been shown to inhibit the PANX1 channels in other systems mentioned above, predicted using databases such as starBase, Targetscan and miRanda, or even synthetic [133-135]. The changes in factors such as NLRP3, ATP, K+ and Ca2+ provide compelling evidence of the activity of the PANX1 channel. A multisensor-integrated organs-on-chips platform designed by researchers at Harvard Medical School has huge potential for application in automated, dynamic, and continual in situ monitoring of these factors [136]. Moreover, TEER-MEA chips designed by Ingbe et al. can monitor the membrane potential and transmembrane potential of cardiomyocytes in real-time. This technology could be leveraged to monitor the current changes associated with the PANX1 channel on nerve cells to analyze its activity [137].

## 5. Conclusion and Outlook

Given the limited time window for acute ischemic stroke treatment and its high mortality and disability rates, a great deal of work has been done in treating acute ischemic diseases [138]. The past few years have witnessed unprecedented advances with effective interventions designed for acute ischemic stroke to extend the time window for treatment. The central and even peripheral inflammatory response after acute ischemic stroke has gradually become a research hotspot since it acts throughout the physiological mechanisms of acute ischemic stroke and may compensate for the lack of existing therapeutic approaches [139]. Interestingly, it has been shown that activation of PANX1 channels in brain resident innate cells (microglia and astrocytes) is the first step in the inflammatory response after acute ischemic stroke. The PANX1 channels are also considered to be a bridge between inflammatory cells and the vascular wall, and their released purinergic signals are involved in the migration of inflammatory cells to the site of ischemia after ischemic stroke and cause secondary damage[140-142]. Moreover, the PANX1 channel, as an upstream molecule of the PANX1-NLRP3 signaling axis, regulates many inflammatory factors in acute ischemic stroke. These factors corroborate the strong clinical value of the PANX1 channel in the treatment of inflammation-mediated acute ischemic stroke, which indicates that it can be a potential target for the treatment of acute ischemic stroke.

Herein, we summarized relevant studies on inflammation mediated by the PANX1 channel in acute ischemic stroke and described the organoid-on-a-chip technology, which could be used to search for miRNAs that could highly target the PANX1 channel to achieve personalized precision therapy for patients with acute ischemic stroke at the genetic level. In addition, exosomes carrying target miRNAs into the human body can successfully cross the BBB and efficiently reach the ischemic site, paving the way for the clinical transformation of targeted PANX1 channels in treating acute ischemic stroke. Although our current framework for screening miRNAs that target the PANX1 channel using brain-organoid-on-a-chip is still rudimentary, it has great application potential. More efforts should be put into this aspect of research to promote breakthroughs in inhibitors of PANX1 channels to provide new options for reducing the inflammatory response of acute ischemic stroke damage. Further research on PANX1 channels in acute ischemic stroke is warranted for developing new drugs targeting PANX1 channels and their subsequent clinical translation.
